# Screening for Depression in Hospitalized Pediatric Patients

**Published:** 2014

**Authors:** Mohammad-Reza ESMAEELI, Reza ERFANI SAYAR, Ali SAGHEBI, Saghi ELMI, Shagheyegh RAHMANI, Sam ELMI, Akram RABBANI JAVADI

**Affiliations:** 1 Pediatric Nephrology Department, Mashhad University of Medical Sciences, Mashhad, Iran; 2 Anesthesiology Department, Pain Clinic, Mashhad University of Medical Sciences, Mashhad, Iran; 3 Psychiatry Department, Shiraz University of Medical Sciences, Shiraz, Iran; 4 Pediatric Department, Mashhad University of Medical Sciences, Mashhad, Iran; 5 Research Center for Patient Safety and Health Quality, Mashhad University of Medical Sciences, Mashhad, Iran; 6 MSc of Mental Health Nursing, Mashhad University of Medical Sciences, Mashhad, Iran

**Keywords:** Depression, Hospitalization, Pediatric patients

## Abstract

**Objective:**

In chronically ill children who are hospitalized, many mood changes occur. For example, in children with cancer or renal failure, prolonged hospitalization and chemotherapy can lead to depression. With the improved survival of childhood malignancies, the effect of treatment on child’s psychosocial well-being becomes increasingly relevant. In this study, we examined the prevalence of depression in hospitalized children with chronic and acute conditions in Dr Sheikh Pediatrics Hospital in Mashhad.

**Materials & Methods:**

After receiving the approval from the Ethics Committee of Mashhad University of Medical Sciences, we did this cross-sectional descriptive study, from April to June 2012 in Dr Sheikh Pediatric Hospital in Mashhad. Ninety children, between 8 to 16 years, were screened for depression. The sampling method was census. Children with a history of depressive or other mental disorders were excluded.

Three groups of children (children with chronic renal disease, malignancy, and acute disease) were evaluated for depression using standard Children Depression Inventory Questionnaire (CDI). Two specifically trained nurses filled out the questionnaires at patients’ bedside under the supervision of a psychiatrist. Depression scores were then analyzed by SPSS software.

**Results:**

Of 90 children, 43(47.7%) were male and 47(52.2%) were female. The Children’s mean age was 11±2.3 years, and the mean length of hospitalization was 8±5.3 days.

Depression was detected in various degrees in 63% of patients (N=57), and 36.6% of children (N=32) had no symptoms of depression. Severe depression was not seen in any of the patients with acute illness. More than half of patients with cancer and chronic kidney disease had moderate to severe depression.

There was a significant statistical relationship between the duration of illness and severity of depression. There was also a significant correlation between severity of depression and frequency of hospitalization. Children who had been hospitalized more than 3 times in the previous year, experienced more severe levels of depression.

We also found a significant correlation between pubertal age and severity of depression in patients with cancers and chronic renal failure.

**Conclusion:**

Children who are hospitalized due to chronic conditions are at a higher risk for mood disorders in comparison with the ones with acute conditions. It is therefore advisable to consider more practical plans to improve the care for hospitalized children’s mental health.

## Introduction

People with depressive disorders have depressed mood or loss of interest or pleasure in usual activities. The typical symptoms of major depression in a young person include:1) Emotional changes: such as feelings of unhappiness, moodiness and irritability, emptiness or numbness, tearfulness or frequent crying, feelings of worthlessness and guilt, sadness and/or hopelessness, loss of interest and pleasure in activities that one once enjoyed, fatigues, lack of energy and motivation, and feeling worried or tense; 2) Cognitive changes: 

difficulty concentrating and making decisions, negative body image and low self esteem, being self-critical and self-blaming, having dark and gloomy thoughts, such as thoughts of suicide or death; 3) Behavioral changes: 

low attention to personal hygiene and appearance, decreased participation with peers and normally enjoyed activities, self harm or deteriorated self-care or promiscuity, avoidance of family interactions and activities, withdrawn behavior such as clearly more time spent alone; and 4) Physical changes: loss of appetite and weight (but sometimes people “comfort eat” and put on weight), either trouble sleeping, or over-sleeping and staying in bed most of the day, decreased libido, restlessness and agitation, unexplained aches and pains (1,2).

Children’s mood is very vulnerable in response to stressors. In chronically ill children who are hospitalized, many mood changes occur. For example, cancer or renal failure, prolonged hospitalization, and chemotherapy can lead to depression in children. Symptoms of anxiety and depression were even seen among parents of children with chronic diseases (3). On the other hand, depressive symptoms in children of cancerous parents persist over time and should be identified soon (4).

With the improved survival of childhood malignancies, the effect of treatment on child’s psychosocial wellbeing becomes increasingly relevant (5). 

Psychiatric disorders are common in children with endstage renal disease (6,7). Especially, hemodialysis and renal transplantation have serious effects on the quality of life (8,9).

In this study, we examined the prevalence of depression in hospitalized children with chronic and acute conditions in Dr Sheikh Hospital in Mashhad.

## Materials & Methods

After receiving the approval from the Ethics Committee of Mashhad University of Medical Sciences, we did this cross-sectional descriptive study, from April to June 2012 in Dr Sheikh pediatric Hospital in Mashhad. Ninety children, with the age range of 8 to 16 years were screened for depression. The sampling method was census. Children with a history of depressive or other mental disorders were excluded. Thirty children had chronic renal failure, undergoing hemo-dialysis, and 30 were suffering from different types of cancer, undergoing chemotherapy and radiotherapy. We randomly selected 30 children with acute conditions, hospitalized for other reasons such as pneumonia, gastroenteritis, etc. At first, the study was explained to the participants and after obtaining their consents, demographic characteristics, such as age, frequency and duration of hospitalization, region of residence, and socioeconomic status were recorded. None of the patients had any history of psychological / psychiatric problems, especially depression, in the past. Also, none of them had any serious problems in their families and had never experienced any major quarrels between their parents in their lives. Three groups of children (children with chronic renal disease, malignancy, and acute disease) were evaluated for depression using standard Children Depression Inventory questionnaire (CDI). Two nurses who had master’s degree in psychology and were specifically trained in a two-hour session for CDI questionnaire, as well as an expert psychiatrist who supervised these nurses, filled out the questionnaires at patients’ bedside. Depression scores were then analyzed by SPSS software. CDI questionnaire has 27 questions and each patient can score between 0 to 54 (because each question has scores of 0, 1, or 2). Based on the total score, the children were divided into 4 groups:

1- No depression (score: 0-4), 2-Nild depression (score: 4-8), 3-Moderate depression (score: 8-12), 4-Severe depression (score: more than 12). Data were analyzed using the non-parametric tests of Kolmogorov-Smirnov and Mann-Whitney.

## Results

A total of 90 patients were studied, 43 of whom were male (47.7%) and 47 were females (52.2%). The mean age of the patients was 11±2.3 years and the mean length of admission was 8±5.3 days.

Eighty percent of patients (N=72) lived in Mashhad –the capital of Khorasan province- and its suburbs, and the socioeconomic status was average in 62% of the patients and their families (N=56).

Depression was detected in various degrees in 63% of patients (N=57), and 36.6% of children (N=32) had no degree of depression. Severe depression was not seen in any of the patients with acute illness. More than half of patients with cancer and chronic kidney disease had moderate to severe depression.

The presence and degree of depression in children are presented in Table 1.

**Table 1:** Presence and Degree of Depression

Diagnosis	Without depression	Mild depression	Moderate depression	Severe depression
Acute illness	23 (76%)	0	7 (25%)	0
Cancers	3 (10%)	0	8 (26.6%)	19 (63%)
Chronic renal failure	7 (21%)	3 (10%)	3 (10%)	17 (51%)
Total	33 (36.6%)	3 (3%)	18 (20%)	36 (40%)

More than 50% of children with severe depression had been suffering from chronic diseases from 1.5 years before the study. There was a significant statistical relationship between the duration of illness (chronicity) and severity of depression (p=0.003). There was also a significant correlation between severity of depression and frequency of hospitalization. Children who had been hospitalized more than 3 times in the last year, experienced more severe levels of depression (p=0.007). 

Children who were hospitalized more than 5 days experienced depression more than the ones who were hospitalized less than 5 days (p=0.045). 

We also found a significant correlation between pubertal age and severity of depression in patients with cancers and chronic renal failure (p=0.006). 

25 children (52.7%) had moderate to severe depression around the age of puberty.

**Table:** 

Duration	Less than 1 year	1 to 1.5 years	1.5 to 3 years	3 to 5 years	More than 5 years	Total
Severity						
No depression	(80%)8	(10%)1	0	(10%)1	0	(100%)10
Mild depression	(33.3%)1	(33.3%)1	0	(33.3%)1	0	(100%)3
Moderate depression	(36.3%)4	(54.5%)6	(9%)1	0	0	(100%)11
Severe depression	(11.1%)4	3(8%)	(5%)2	(16%)6	(58%)21	(100%)36
Total	(100%)60	(100%)21	(100%)3	(100%)11	7(100%)	

## Discussion

It is not easy to find a direct correlation between hospitalization and children’s mental health status. There are several particular contributing factors, such as severity of pain, degree of disability in activities, and quality of family support. 

Several strategies were undertaken to identify patients who are at an increased risk for developing a depressive disorder, because of the relationship to major depressive disorders (MDD) and their deleterious effect on patients’function. Depressive disorders are one of the most common causes of psychiatric disability in the society. 

The present study is one of the first studies in Iran on depressive symptomatology in hospitalized children. 

The aim of this study was to assess the prevalence of depression and its risk factors. In our study, 63% of children had some degrees of depression. More than 50% of children with severe depression had been suffering from chronic diseases from 1.5 years before the study. Depression might deteriorate with pain, disabilities, long-term or several repeated hospitalization, and puberty.

Laffond’s study in 2012 in France showed that 38% of children with craniopharyngioma (mean age of 6 years) suffered from depression, but there was no comparison with other patient groups in this study (10). Chung in USA showed that 65% of patients with tuberosclerosis experienced some degrees of mood disorders, which is in accordance with our findings. Mean chronicity of their illness was 20 months (11). Kinahan et al.’s study in 2007 on the life expectancy of children with cancer in America showed that many of them suffered from emotional and mood disorders due to different causes, such as hair loss, prolonged hospitalization, and treatment process. 

This significantly worsened their quality of life (12). Adduci et al.’s study in Italy in 2012 demonstrated that the incidence of mood disorders and anxiety in children with brain tumor has a direct correlation with the quality of relationship between children and their parents and with parental supports (13).

Szabo et al. studied 108 children aged 7 to 17 years (mean, 8 years) and with the admission duration of 0.5 to 14 years in Hungary. Their patients were divided into two groups of asthma and renal failure; 50% of female patients were depressed according to Beck indicators. There was no significant difference in the severity of depression between the two groups (14). 

Arabiat et al. in 2012 in Jordan compared the prevalence of depression in 58 children with cancer, 56 children with chronic disease and 64 healthy children according to Arabic version of CDI. There was no significant difference in CDI scores between the 3 groups; 20.68% of depressed children suffered from cancer (15). Their results are in contradiction with ours. Most studies confirm the results obtained in our study. It seems that poor prognosis diseases in childhood could lead to more severe mental disorders, because it can affect both children and their parents. Therefore, children with chronic diseases need family support to feel better and have a better quality of life.

It seems that the presence of emotional disorders such as depression and anxiety can aggravate underlying medical conditions or deter the course of their improvement or response to treatment. Therefore, prompt diagnosis and early treatment of mood disorders or anxiety improves the quality of life and accelerates the treatment process of medical conditions. On the other hand, therapy-related symptoms could be a beneficial indicator in screening the pediatric patients with psychosocial distress or at risk for depression (16). 

It is therefore advisable to consider more practical plans to improve the care for hospitalized children’s mental health. Plans such as employment of expert pediatric psychologists and educated consultants in pediatric oncology and dialysis wards could be considered as a few examples. They can provide counseling and psychotherapy for patients, their parents and even their visitors. Although cheerful colors, playing rooms, and various entertainments were prepared for children, and several psychiatrists are available in Dr Sheikh Hospital; it seems not to be enough, and a more focused attention is needed in this regard. Identifying vulnerable children with chronic conditions and providing different psychosocial interventions for them and their family are crucial.

One of our limitations was not evaluating parents’ mental health and support, which could have a great effect on children’s psychopathology. 

**In conclusion, **children who are hospitalized due to chronic conditions are at a higher risk for mood disorders in comparison with the ones with acute conditions. 
